# Comment on “Deliberate self‐harm in adolescents with OTC‐related psychiatric disorders: A study of prevalence and associated factors”

**DOI:** 10.1002/pcn5.70295

**Published:** 2026-02-10

**Authors:** Sushma Narsing Katkuri, Varshini Vadhithala, Sachin Kumar, Sushma Verma, Jeffrin Reneus Paul

**Affiliations:** ^1^ Department of Community Medicine Malla Reddy Institute of Medical Sciences Malla Reddy Vishwavidyapeeth Hyderabad Telangana India; ^2^ Dr. D. Y. Patil Medical College, Hospital and Research Centre Dr. D. Y. Patil Vidyapeeth (Deemed‐to‐be‐University) Pune Maharashtra India; ^3^ Faculty of Pharmaceutical Sciences Graphic Era Hill University Dehradun India; ^4^ Centre for Promotion of Research Graphic Era Deemed University Dehradun India; ^5^ Department of Pharmaceutics Noida Institute of Engineering & Technology (Pharmacy Institute) Greater Noida India; ^6^ Saveetha Medical College and Hospital, Saveetha Institute of Medical and Technical Sciences Saveetha University Chennai India

Dear Editor,

We read with great interest the recent article by Matsumoto et al. entitled “*Deliberate self‐harm in adolescents with OTC‐related psychiatric disorders: A study of prevalence and associated factors*,” published in *Psychiatry and Clinical Neurosciences Reports*.^1^ The authors should be commended for addressing the increasingly important and underexplored intersection between over‐the‐counter (OTC) drug misuse and deliberate self‐harm (DSH) among adolescents in Japan.

Using data from the 2024 Nationwide Psychiatric Hospital Survey, the authors report an exceptionally high prevalence of past‐year DSH (82.8%) among adolescents with OTC‐related psychiatric disorders. Notably, self‐harm was associated with female sex, lower educational attainment or ongoing school enrollment, and subthreshold “harmful use,” while no significant associations were observed with dependence severity, recent OTC use, psychiatric comorbidity, or utilization of addiction treatment resources. These findings challenge conventional addiction‐centered frameworks and highlight the need for alternative clinical perspectives. Beyond summarizing the original findings, this commentary seeks to reframe adolescent OTC‐related self‐harm away from an addiction‐severity paradigm and toward a developmental vulnerability and suicide‐prevention framework, with direct implications for clinical assessment and public health intervention.

One important methodological issue concerns the broad operationalization of DSH in the survey design. As acknowledged by the authors, the instrument did not distinguish between non‐suicidal self‐injury, intentional overdose, and suicide attempts. This lack of differentiation may have inflated prevalence estimates, particularly because intentional overdose may simultaneously represent both substance misuse and self‐harm. Prior evidence indicates that method‐specific distinctions are clinically meaningful, given differences in intent, lethality, and recurrence risk across self‐harm behaviors.^2^, ^3^ Future nationwide psychiatric surveys would benefit from explicitly distinguishing non‐suicidal self‐injury, self‐poisoning with suicidal intent, and suicide attempts, thereby improving clinical interpretability and risk stratification.

The observation that adolescents classified under harmful use—rather than dependence—exhibited the highest risk of DSH is nevertheless clinically informative. However, given the cross‐sectional nature of the study, causal direction cannot be inferred. Alternative explanations, including reverse causality (whereby underlying self‐harm vulnerability precedes OTC misuse) or unmeasured confounding related to psychiatric severity or psychosocial stress, must be considered. Even so, this pattern suggests that OTC misuse in adolescence may often function as a maladaptive coping strategy rather than a manifestation of entrenched addiction. This interpretation is consistent with developmental neuroscience literature describing heightened impulsivity, emotional reactivity, and immature executive control during adolescence, which may lower thresholds for self‐harming behaviors when psychoactive substances are readily accessible.^4^

Educational status emerging as an independent correlate of self‐harm also warrants cautious interpretation. In the original dataset, variables such as educational attainment and school enrollment may reflect heterogeneous constructs, including developmental stage, academic difficulty, school refusal, or broader psychosocial adversity. As such, these findings should not be interpreted as indicating a direct causal relationship between education and self‐harm.^5^ Given that the majority of affected individuals were still enrolled in school, these findings underscore the importance of school‐based identification, prevention, and collaboration between educational institutions and mental health services.

From a clinical and public health standpoint, the authors' conclusion that abstinence‐oriented addiction treatment alone may be insufficient is well supported. Harm‐reduction–informed, suicide‐prevention–focused interventions—particularly those integrated into educational and community settings—may be more appropriate for this population.^6^ Longitudinal studies incorporating method‐specific self‐harm data and school‐related psychosocial variables would further clarify causal pathways and inform targeted interventions.

In summary, Matsumoto et al. provide compelling evidence that adolescents with OTC‐related psychiatric disorders represent a distinct, high‐risk group in whom self‐harm vulnerability cannot be adequately addressed through traditional addiction frameworks alone. Their work highlights the urgent need to integrate suicide prevention, harm reduction, and educational collaboration into clinical care for adolescent OTC misuse.

## AUTHOR CONTRIBUTIONS

**Sushma Narsing Katkuri**: Methodology; writing—original draft; writing—review and editing. **Varshini Vadhithala**: Writing—original draft; writing—review and editing. **Sachin Kumar**: Conceptualization; validation; writing—review and editing. **Sushma Verma**: Writing—original draft; writing—review and editing. **Jeffrin Reneus Paul**: Validation; writing—review and editing.

## CONFLICT OF INTEREST STATEMENT

The authors declare no conflicts of interest.

## ETHICS APPROVAL STATEMENT

Not applicable.

## PATIENT CONSENT STATEMENT

Not applicable.

## CLINICAL TRIAL REGISTRATION

Not applicable.

## APPROVAL OF THE RESEARCH PROTOCOL BY AN INSTITUTIONAL REVIEWER BOARD

This study did not involve human participants or animal subjects requiring institutional review board or animal ethics approval.

## ANIMAL STUDIES

Not applicable.

## Data Availability

Data sharing is not applicable to this article as no datasets were generated or analyzed during the current study.
